# Clinical progress note: Rubella

**DOI:** 10.1002/jhm.70169

**Published:** 2025-09-12

**Authors:** Adam E. Gailani, Walter Dehority, Sophie E. Katz

**Affiliations:** ^1^ Division of Pediatric Infectious Diseases Vanderbilt University Medical Center Nashville Tennessee USA

## Abstract

Rates of rubella infection and congenital rubella syndrome decreased significantly since the introduction of the rubella vaccine in 1969. Endemic rubella was declared eliminated in the United States in 2004, and since 2012, all rubella cases in the United States have been associated with infections acquired abroad. With vaccine rates falling worldwide and outbreaks of vaccine preventable diseases increasing, it is important for clinicians to be prepared to recognize and manage diseases they may have never seen before, including rubella. This article reviews the clinical manifestations, complications, diagnosis, management, and prevention of acute rubella infection and congenital rubella syndrome.

## Visual Abstract

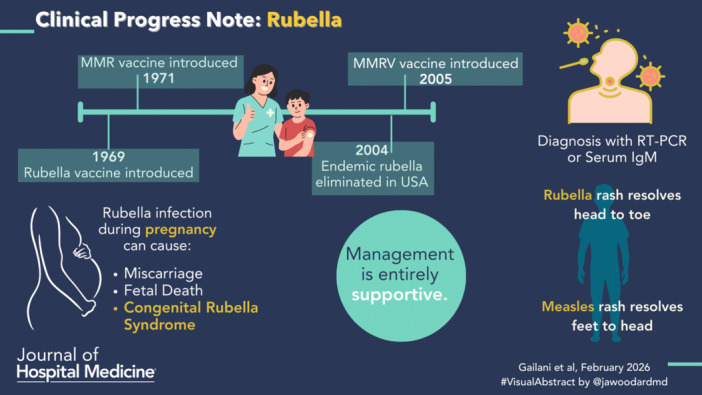

## INTRODUCTION

Vaccine hesitancy, and the subsequent re‐emergence of vaccine preventable diseases, is one of the greatest modern threats to global health and the progress made during the 20th century against infectious diseases. One of these diseases is rubella, a viral infection which is no longer endemic in the United States but is at risk of re‐emergence, and is the focus of this article. Models estimating disease re‐emergence in the face of declining immunization rates in the United States^1^ paint a sobering picture of the potential burden these diseases could inflict if recent trends of decreasing vaccination rates continue and lead to significant gaps in coverage. A large measles outbreak in 2025, originating in Texas and primarily affecting unimmunized individuals, demonstrates the risk such outbreaks of vaccine preventable diseases pose to communities and public health agencies.^2^ With the danger for once eradicated diseases to return to the United States, it is vital for clinicians to know their clinical manifestations, complications, prevention strategies, and management. Information in this article was sourced from society, organization, and governmental agency guidelines, as well as from a focused literature search in PubMed for papers on rubella diagnosis, management, and prevention using keywords including “rubella,” “congenital rubella,” and “rubella immunization.”

## VACCINE IMPACT

**Figure 1:**
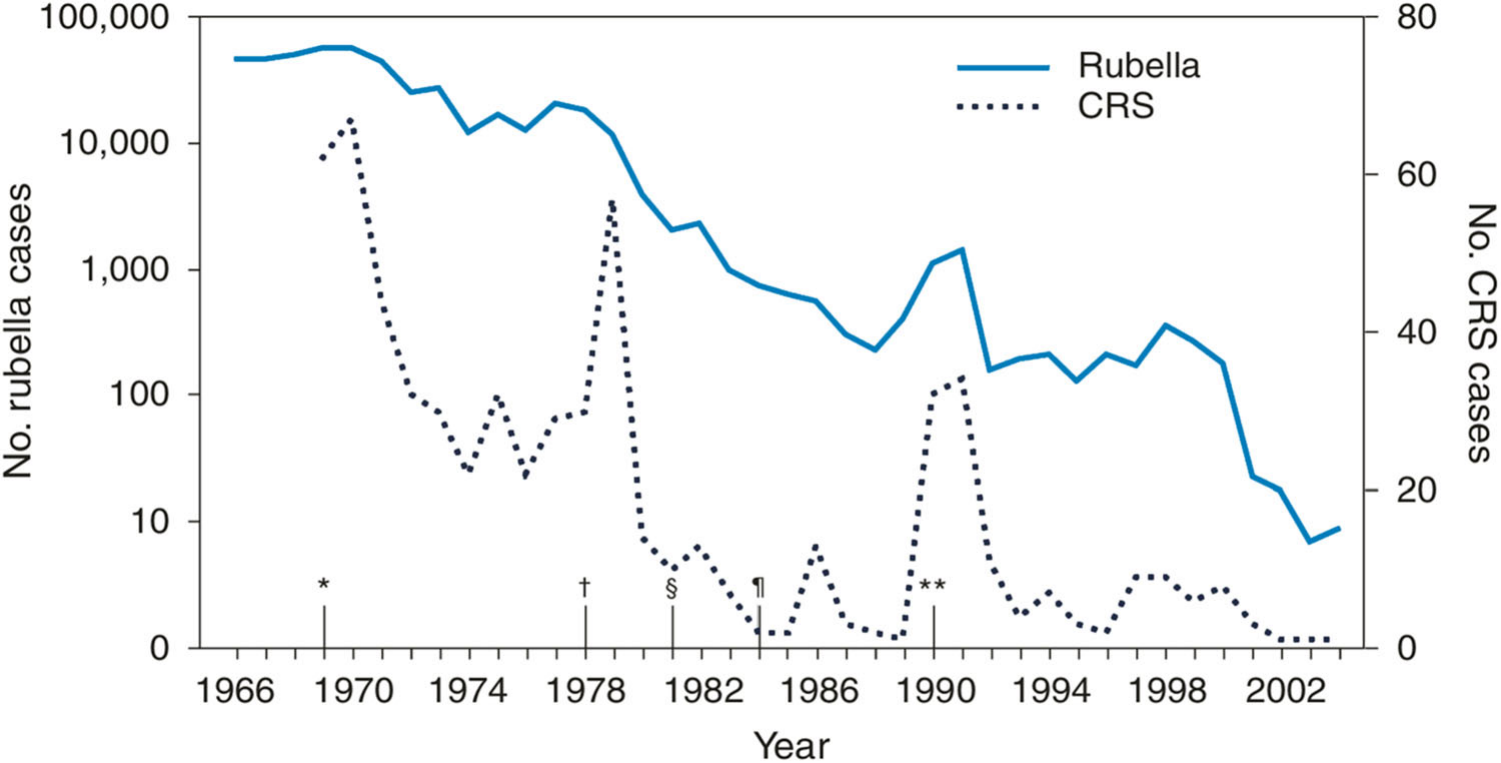
Number of reported cases of rubella and congenital rubella syndrome (CRS), by year, and chronology of rubella vaccination recommendations by the Advisory Committee on Immunization Practices—United States, 1966–2004. Copied from CDC MMWR, public domain.^6^ *1969—First official recommendations are published for the use of rubella vaccine. Vaccination is recommended for children aged 1 year to puberty. †1978—Recommendations for vaccination are expanded to include adolescents and certain adults, particularly females. Vaccination is recommended for adolescent or adult females and males in populations in colleges, certain places of employment (e.g., hospitals), and military bases. ^§^1981—Recommendations place increased emphasis on vaccination of susceptible persons in training and educational settings (e.g., universities or colleges) and military settings, and vaccination of workers in health‐care–settings. ^¶^1984—Recommendations are published for vaccination of workers in daycare centers, schools, colleges, companies, government offices, and industrial sites. Providers are encouraged to conduct prenatal testing and postpartum vaccination of susceptible women. Recommendations for vaccination are expanded to include susceptible persons who travel abroad. **1990—Recommendations include implementation of a new two‐dose schedule for measles‐mumps‐rubella vaccine. 2004—End of graph. Rubella is declared eradicated in the United States (lack of continuous transmission for 12 months).

A vaccine for rubella was first introduced in the United States in 1969, followed by the combination MMR (measles, mumps, and rubella) vaccine in 1971 and the combination MMRV (measles, mumps, rubella, and varicella) vaccine in 2005.^3^ Rubella vaccination in the United States is currently administered as part of the MMR or MMRV vaccine, and is given as a two‐dose series at ages 12–15 months and 4–6 years.^4^ People born in and after 1957 should receive at least one dose of a rubella containing vaccine if they do not have evidence of immunity (IgG levels ≥ 10 IU/mL).^5^ Rates of rubella infection and congenital rubella syndrome decreased significantly in the United States following the initial introduction of rubella vaccination in 1969,^6^ Figure 1. Endemic rubella was eliminated in the United States in 2004, and since 2012 all rubella cases in the United States have arisen from infections acquired abroad.^3^ Cohort studies evaluating the effectiveness of rubella immunization have shown vaccine effectiveness ranging from 78.6% to 89% for preventing rubella infection.^7^, ^8^

A Cochrane review demonstrated the safety and efficacy of MMR and MMRV vaccines.^7^ Current data shows no risk of any serious adverse events with rubella vaccination. An association between certain MMR vaccines and aseptic meningitis exists, as well was an association between MMR/MMRV vaccines and febrile seizures. An association is also noted with idiopathic thrombocytopenic purpura (ITP), however rates of ITP following natural infection with measles, mumps, or rubella are twice as high as following vaccination.^7^

MMR and MMRV are live virus vaccines, and as such are contraindicated in pregnant individuals and those with severe immunodeficiencies.^4^ However, studies on the administration of MMR vaccines in specific populations of solid organ transplant patients demonstrate these vaccines are well tolerated.^9^ Such data may lead to changes in guidelines and contraindications for MMR and MMRV vaccines in the future.

Individuals may report opposition to MMR/MMRV vaccination on various religious grounds due to the use of porcine gelatin and preparation of the rubella vaccine in cell lines derived from fetal tissue. However, no major world religious doctrine explicitly prohibits vaccination for its members. Interestingly, the Catholic Church's Pontifical Academy for Life issued a statement affirming the use of such vaccines if there is no alternative, to avoid the risk of fetal morbidity and mortaility in one's own children and the population as a whole. Their statement highlights the remoteness of the connection to the initial abortion from which the cell lines were derived, but does call for vaccines to be developed without using aborted fetal cells.^10^

## CLINICAL MANIFESTATIONS/COMPLICATIONS

**Figure 2:**
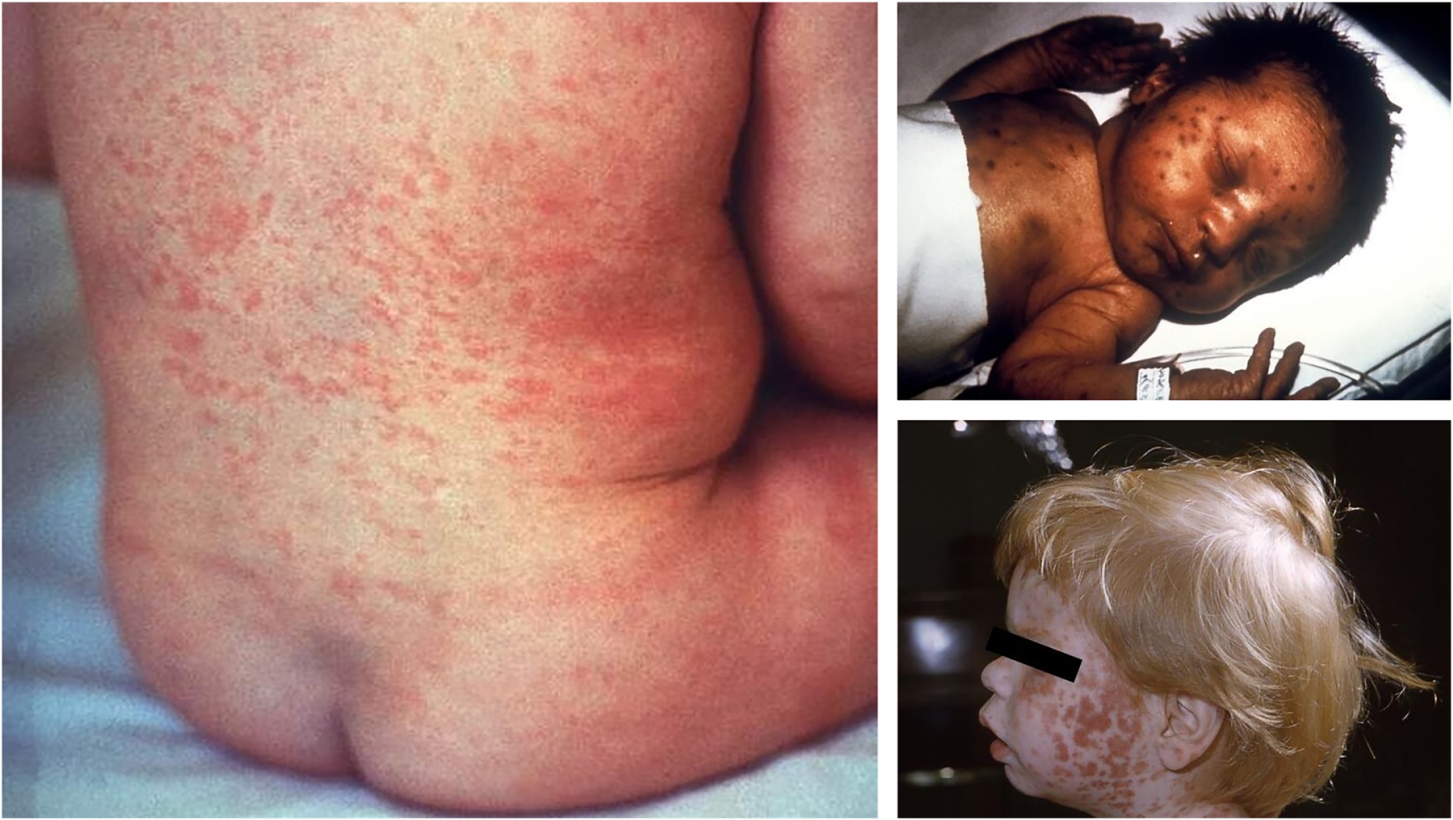
Rashes of Rubella. Left and bottom right: typical rash associated with an acute rubella infection. Upper right: infant with congenital rubella syndrome and “blueberry muffin” rash.^11^ All images are in the public domain.

The classic manifestation of an acute rubella infection is an erythematous rash that starts on the face and spreads down the rest of the body (Figure 2), similar to measles, and lasts up to 3 days. Other symptoms may be present 1–5 days before the rash appearing, and include fever, cough, rhinorrhea, headache, conjunctivitis, myalgias, arthralgias, fatigue, and lymphadenopathy. Cases can be subclinical, especially in adults where up to 50% of those infected can be asymptomatic. Rubella virus, both vaccine and wild strains, has been identified in granulomas, often at the site of previous, remote rubella vaccination, of patients both with and without immunodeficiencies. Research into these cases has shown that rubella may be a causative agent in some cases of idiopathic granulomatous dermatitis.^12^ The incubation period for acute rubella infection is 2–3 weeks, and those with rubella are most infectious in the week before and the week after the appearance of the rash.^5^, ^13^, ^14^

Given the similarity between measles and rubella, which is also known as German measles, it is important to know which features distinguish them from one another. While both rashes will appear on the head and spread down to the rest of the body, rubella's rash will resolve from head to toe while measles's rash resolves from the feet up. The lymphadenopathy associated with rubella is often of the posterior auricular or suboccipital chains, which is not the case in measles infections. Koplik spots (small red spots with blue/white centers on the oral mucosa) are seen in the prodromal period of measles, but not rubella infections. While those with rubella are infectious a full week before and after the appearance of the rash, those with measles are only infectious during the 4 days before and after the appearance of the rash.^5^, ^15^

Before the advent and implementation of rubella immunization, as many as 4 out of 1000 live births worldwide developed congenital rubella syndrome, making it one of the more significant complications of rubella infection.^14^ Infection with rubella during pregnancy can lead to miscarriage, fetal death, or a number of congenital anomalies if the fetus survives, which are collectively known as congenital rubella syndrome. Between 1962 and 1965, rubella caused 11,250 fetal deaths, 2100 neonatal deaths, and 20,000 cases of congenital rubella syndrome in the United States alone.^6^ Anomalies can involve multiple different organ systems. Ophthalmologic involvement can manifest as cataracts. Cardiac manifestations include patent ductus arteriosus and peripheral pulmonary artery stenosis. Sensorineural hearing loss, microcephaly, developmental disabilities, behavioral disorders, and autism can also occur secondary to CNS involvement. Manifestations noted in utero and shortly after birth include growth restriction, hepatosplenomegaly, thrombocytopenia, dermal erythropoiesis (“blueberry muffin” rash, Figure 2), interstitial pneumonitis, and radiolucencies in the metaphysis of bones on X‐ray. Babies with congenital rubella syndrome may only develop some of these manifestations and may be initially asymptomatic at birth in milder forms of the syndrome.^5^, ^13^, ^14^ Several other complications may occur following rubella infection in those who are not pregnant. These include arthritis, which is more common in adult women, in whom up to 70% may be affected.^13^, ^14^ Encephalitis occurs in 1 in 6000 cases and thrombocytopenia in 1 in 3000 cases.^5^

## DIAGNOSIS

Diagnosis of an acute rubella infection involves either the detection of RNA by reverse transcriptase‐polymerase chain reaction (RT‐PCR), or by serology.^5^, ^13^ RT‐PCR testing can be performed on nasopharyngeal swabs, throat swabs, and/or urine samples. Nasopharyngeal or throat swabs should be obtained as soon as rubella is suspected, preferably within 3 days of rash onset, but can be useful up to 7 days after rash onset. Urine can be tested within 7 days of rash onset as well. Serum immunoglobulin (Ig) M detection can be used to diagnose acute rubella infection and is typically present 4–5 days after rash appearance and persists up to 30 days after rash onset. Detection of IgG is typically used to assess evidence of immunity to rubella, such as testing during pregnancy, and should become positive 2 weeks after immunization. A fourfold rise in IgG titers between acute and convalescent serum samples indicates an acute infection. Serum IgM and IgG should be collected on pregnant people with unknown immunity to rubella, if those pregnant people are exposed to rubella. If testing shows they are not immune, they should be retested again in 2–3 weeks and 6 weeks, along with frozen serum from the initial exposure to determine if there is a rise in titers consistent with acute infection.^5^

Testing for congenital rubella syndrome can be accomplished with the same tests noted above.^5^ Positive RT‐PCR samples from the nasopharynx, throat, and/or urine indicate congenital infection. Detection of rubella IgM within the first 6 months of life or a rise in rubella IgG in the 7–11 month‐old range, after maternal antibodies have waned, also indicate congenital infection.

Viral culture can be used to isolate rubella viruses from patient samples, but is typically not recommended as the primary method of diagnosing rubella as it takes much longer to perform compared to RT‐PCR or antibody testing and requires specialized culture methods.^5^, ^13^

The CDC provides a guidance sheet on commercially available rubella tests and recommendations on appropriate use and timing of the tests.^13^

## MANAGEMENT

There is no specific antiviral treatment or other therapy for rubella infections, acute or congenital. Management is entirely supportive.^5^, ^13^

Children with acute rubella infection should be excluded from school or childcare for 7 days from the onset of the rash. Infants with congenital rubella infection are considered infectious and droplet precautions are recommended while they are admitted to the hospital until they are 1 year of age, unless negative RT‐PCR testing is obtained on two tests, 1 month apart, and the infant is over 3 months of age. In an outbreak setting, children who are not immune to rubella (at least one rubella immunization after 1 year old), should be excluded from school or childcare for 21–23 days after the onset of rash in the most recent case.^5^, ^13^ The administration of immunoglobulin for secondary prophylaxis in an exposed person is not recommended, and administration of rubella vaccine after exposure has not been shown to prevent acquisition of rubella but should still be strongly considered as it will confer protection for future exposures.^5^

## CONCLUSION

With vaccine rates falling worldwide, and the recent occurrence of outbreaks of vaccine preventable diseases here in the United States, it is important for clinicians to be prepared to identify and manage diseases like rubella. Benjamin Franklin is famously quoted as saying “an ounce of prevention is worth a pound of cure”. All efforts should be made at the local, national, and global level to promote vaccination and reverse the trends of decreasing vaccination rates seen in many countries. If these trends continue, we may see the return of these vaccine preventable infections, and with them the diseases that ravaged countless previous generations.

## CONFLICT OF INTEREST STATEMENT

The authors declare no conflicts of interest.
